# Editorial: Millets: superfood of the century – challenges and opportunities

**DOI:** 10.3389/fnut.2024.1462567

**Published:** 2024-08-15

**Authors:** Veda Krishnan

**Affiliations:** Division of Biochemistry, ICAR- Indian Agricultural Research Institute, New Delhi, India

**Keywords:** millet, superfood, sustainable agriculture, therapeutics, glycemic control

This Research Topic gathers different contributions highlighting the role of millets, its nutritional profile, sensory attributes, therapeutic benefits, and other agronomic advancements. These approaches allow us to shed light on millets and their functional importance in diet as the emerging super-foods of the 21st century.

The first article of this Research Topic (Prabhakar et al.), highlights that using organic amendments such as compost and green manures can significantly improve soil structure, water retention, and nutrient content, leading to sustainable agriculture practices in finger millet. The field study conducted in 2018 and 2019 compared the effects of chemical fertilizers and farmyard manure (FYM) on two finger millet varieties, MR-1 and MR-6, under four nutrient management practices. The MR-6 variety outperformed MR-1, achieving up to 22.6% higher yield. Substituting FYM for chemical fertilizers, either partially or fully, resulted in better growth, yield, and nutrient uptake. The most effective practice was 100% RDN as FYM, followed by 50% RDF + 50% RDN as FYM. Higher FYM application improved soil properties significantly, boosting organic carbon by 25% and available nutrients. Organic manure substitution enhances yields, economic returns, and soil health.

The other six articles outlined the contributions and implications focused on the nutritional profile of millets, and sensory attributes and positioned them as formidable allies in the fight against non-communicable diseases (NCDs) such as diabetes, hypertension, and cardiovascular diseases, which are increasingly prevalent in India. Richness in dietary fiber, essential minerals, and antioxidants, which collectively contribute to improved metabolic health and reduced disease risk, were discussed. The six original research articles in this Research Topic speak to three major themes related to therapeutic applications:

1. *Variation in the Nutrient Content of Different Genotypes and Varieties of Millets, Studied Globally (Anitha Rajendran, et al.)*.

A global systematic review of studies reveals notable variations in the nutritional composition of different millet types, such as pearl millet, finger millet, and foxtail millet. For example, some finger millet varieties are exceptionally rich in calcium and iron, which are crucial for combating micronutrient deficiencies in vulnerable populations. Pearl millet stands out for its high protein and essential amino acid content, while foxtail millet is abundant in dietary fiber and B vitamins. These differences highlight millet's ability to meet diverse dietary needs. It is essential for breeders, nutritionists, and policymakers to understand these distinctions to promote millet as a staple food. Selecting and cultivating the most nutrient-dense millet varieties can significantly enhance food security and improve nutritional health, especially in regions facing malnutrition and food shortages.

2. *Sensory and Nutritional Evaluation of Nine Types of Millet Substituted for Polished White Rice in Select Indian Meal Preparations (Anitha, Arjun et al.)*.

A study evaluating the substitution of nine types of millet for polished white rice in traditional Indian dishes provides valuable insights. The findings indicate that millets not only enhance the nutritional value of meals but also offer unique flavors and textures that can appeal to diverse palates. For example, replacing rice with millets like barnyard millet or kodo millet in dishes such as biryani or khichdi results in meals that are richer in fiber, protein, and micronutrients while maintaining satisfactory taste and texture. Consumer acceptance is crucial for the success of millets in the mainstream market. Educating the public about the health benefits and culinary versatility of millets, along with efforts to develop and promote recipes that highlight their strengths, can drive the shift toward more nutritious and sustainable food systems.

3. *Cultivating Health: Millets' Potential in Combating Non-Communicable Diseases and Future Research Avenues in India (Bhattacharya)*

Comprehensive reviews suggest that regular consumption of millet can lead to better glycemic control, reduced blood pressure, and lower cholesterol levels. The complex carbohydrates in millets are digested slowly, providing sustained energy release and preventing spikes in blood sugar levels. Furthermore, the high fiber content promotes satiety and helps in weight management, a critical factor in combating obesity, a major risk factor for many NCDs. Despite the numerous health benefits of millets, concerns have been raised regarding the potential goitrogenic effects of certain varieties, particularly pearl millet. Anitha, Upadhyay, Grando et al. addressed this through a systematic review of the existing literature, which reveals a complex relationship between pearl millet consumption and thyroid health. Pearl millet contains goitrogens like thiocyanate, which are substances that can interfere with thyroid function, leading to goiter—a condition characterized by the enlargement of the thyroid gland. Thiocyanate can inhibit iodine uptake by the thyroid gland, especially in populations with marginal iodine intake. However, the evidence is not conclusive, and the risk appears to be context-dependent, influenced by factors such as iodine status, dietary diversity, and overall health. There's also a need for large-scale clinical trials to substantiate these findings and encourage the inclusion of millets in dietary guidelines and public health policies.

Another aspect highlighted is the ability of millet to promote satiety and reduce hunger, which can play a significant role in weight management and the prevention of obesity. A systematic review of studies on the satiating effects of millets reveals that their high fiber and protein content, along with the slow digestibility of their carbohydrates, makes them an effective dietary choice for enhancing fullness and reducing food intake. The consumption of millet leads to a gradual release of glucose into the bloodstream, preventing the rapid spikes and crashes that are typical with refined grains. This steady energy release helps to maintain stable blood sugar levels, prolongs the feeling of fullness, and reduces the likelihood of overeating. Future research should focus on identifying the bioactive compounds in millets that confer these health benefits and developing functional food products that can easily integrate into the modern diet.

## Conclusion

Millets, with their rich nutritional profile and adaptability to various growing conditions, are poised to become the superfood of the century. They offer a sustainable solution to the challenges of food security, health, and environmental sustainability. However, there are hurdles to overcome, including ensuring adequate iodine intake to prevent goiter, improving consumer acceptance through education and culinary innovation, and maximizing their nutritional potential through careful selection and breeding of varieties.

The integration of organic nutrient management, as seen with finger millet in Southern India, underscores the potential of millet to enhance soil fertility and crop yields. Their role in combating non-communicable diseases and promoting satiety further cements their place in a healthy diet. With continued research and strategic promotion, millets can transform global food systems and pave the way for a healthier, more sustainable future.

